# Biosurveillance for invasive insect pest species using an environmental DNA metabarcoding approach and a high salt trap collection fluid

**DOI:** 10.1002/ece3.7113

**Published:** 2021-01-28

**Authors:** Robert G. Young, Yoamel Milián‐García, Jaeju Yu, Erin Bullas‐Appleton, Robert H. Hanner

**Affiliations:** ^1^ Department of Integrative Biology University of Guelph Guelph ON Canada; ^2^ Animal Biosciences University of Guelph Guelph ON Canada; ^3^ Canadian Food Inspection Agency Ottawa ON Canada

**Keywords:** cytochrome *c* oxidase subunit 1, environmental DNA, forest, introduced species, Lindgren funnel traps, molecular identification, plant pest, salt buffer

## Abstract

1. With the increase in global trade and warming patterns, the movement, introduction, and establishment of non‐native insect species has increased. A rapid and effective early detection biosurveillance program to identify species of concern is needed to reduce future impacts and costs associated with introduced non‐native species. One of the challenges facing insect surveillance trapping methods is the sheer volume of individual specimens in the collections. Although molecular identification methods are improving, they currently have limitations (e.g., destructive processing of specimens) and a protocol addressing these limitations can support regulatory applications that need morphological evidence to corroborate molecular data.
2. The novel protocol presented here uses a metabarcoding approach to amplify environmental DNA from a saturated salt solution trap fluid, which retains trap specimens for downstream morphological identifications. The use of a saturated salt solution to preserve specimens in traps addresses issues with the high evaporation rate of ethanol in traps, and public safety concerns with other fluid preservation options with unattended traps in public settings.
3. Using a metabarcoding approach, a 407‐nucleotide segment of the cytochrome *c* oxidase subunit 1 (COI) animal barcode region was successfully amplified from Lindgren funnel trap collection fluids. These traps were placed in forested areas to survey for wood‐boring beetles of regulatory concern. Our results displayed successful amplification of target taxa, including the molecular identification of the Japanese Beetle *Popillia japonica*, a species regulated in Canada. A second species, *Anisandrus maiche*, recently introduced to North America, was identified in every trap. The genus *Lymantria*, which contains numerous species of concern to North American woodlands, was also detected. Also, there were six other species identified of interest due to their potential impacts on native and crop flora and fauna.
4. Our results show how this protocol can be used as an efficient method for the surveillance of insects using a trap with a saturated salt solution and eDNA metabarcoding to detect species of regulatory concern.

## Graphical abstract

A forest survey using Lindgren funnel traps and a metabarcoding approach to amplify environmental DNA from a saturated salt solution trap fluid, which retains trap specimens for downstream morphological identifications. This method amplified DNA which matched more than 1,600 taxa using the COI animal DNA barcode. Of these taxa, there were numerous identified species of concern to Canadian forests.

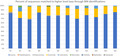

## 1 INTRODUCTION

The volume of global shipping has created massive opportunities for hitchhiking insect pests present on packaging and products, resulting in their movement across large global regions (Perrings, 2016; Westphal et al., 2008). With global warming trends opening new hospitable habitat for non‐native species, there is a need for more surveillance protocols and early detection methods to limit invasive species impacts (Dukes et al., 2009; Stensgaard et al., 2019). The early detection of non‐native insect species is essential to help reduce the impact of introduced species and mitigate the establishment and spread of these species through the invasive framework (Blackburn et al., 2011; Giovani et al., 2020; Richardson et al., 2000). A rapid and effective biosurveillance program needs to be adopted and implemented to identify species of concern and address introduced specimens and populations early (Roe et al., 2019).

Traditional biosurveillance methods that include specimen collections using a variety of trapping methods followed by morphological identification are time‐consuming and costly (Poland & Rassati, 2019). Molecular identification techniques, such as DNA barcoding, have been adopted for use in monitoring to speed identifications and standardize and digitize results (Hebert et al., 2003). The benefits of a DNA barcoding approach as a plant pest surveillance tool to reduce impacts on native crops and native diversity through early detection have been well established (Roe et al., 2019). However, a DNA barcoding methodology still relies on a one‐by‐one specimen approach, which is labor intensive and time‐consuming. In addition, the use of DNA barcoding often occurs at points of suspected introductions such as border inspection sites. Morphological survey results from these sites have established that only about 10% of invasive species were identified prior to establishment (Kenis et al., 2007). This low percentage shows how targeted surveys and a one‐by‐one morphological identification or DNA barcoding approach are not effective at detecting introduced species on a broad scale, an important first step in limiting the impacts of non‐native species.

There is a need to utilize molecular approaches, including high‐throughput sequencing for the biosurveillance of regulated insects recognized as invasive plant pests (Poland & Rassati, 2019; Stensgaard et al., 2019). Moving to a metabarcoding biosurveillance approach can reduce the cost and time associated with traditional one‐by‐one DNA barcoding methods (Cristescu, 2014). While molecular approaches, including DNA barcoding using Sanger sequencing and metabarcoding using high‐throughput sequencing, have been utilized in insect surveillance studies, challenges remain (Deiner et al., 2017; Poland & Rassati, 2019; Watts et al., 2019). There is a need for protocol development to support effective biological metabarcoding research and to further the development of appropriate experimental designs (Makiola et al., 2020; Taberlet et al., 2012; Zinger et al., 2019).

The development of environmental DNA (eDNA) metabarcoding protocols is especially important within the context of the regulation of species by governmental agencies. When detecting taxa, the retention of whole specimens is essential for regulatory purposes to verify suspected presence. Recently eDNA from ethanol in traps has been used in a high‐throughput metabarcoding approach to identify DNA of species while retaining voucher specimens (Hajibabaei et al., 2012; Zenker et al., 2020). The use of ethanol for the preservation of specimens is effective in maintaining DNA for molecular analyses (King & Porter, 2004; Zimmermann et al., 2008). However, ethanol also causes insect specimens to become brittle, can cause changes in soft tissues such as discoloration and variable amounts of morphological changes, and can degrade small‐bodied organisms if maintained in solution at temperatures above freezing for extended periods (King & Porter, 2004; Moreau et al., 2013). In addition to the issues with the preservation of morphological characteristics, the high evaporation rate of ethanol in traps, which are often left in the field for extended periods, and the placement of traps with high concentration ethanol in publicly accessible locations, provides further complications for the use of ethanol in field applications. Here we present a biosurveillance protocol using a saturated salt solution and a next‐generation metabarcoding approach that detects taxa of regulatory interest while maintaining intact specimens and simultaneously addressing concerns with the use of ethanol.

## 2 MATERIALS AND METHODS

### 2.1 Sample collections

To target wood‐boring beetles in the order Coleoptera, Lindgren funnel traps baited with Ethanol Ultra High Release (UHR) Lures (Synergy Semiochemical Corp), were used to survey two Southern Ontario locations, one in Windsor and another in Wheatley (Table 1). Lindgren funnels collection cups were filled with a salt (NaCl) water solution to preserve trapped insect specimens until collection. Saltwater suspension solution was made in the laboratory before field deployment by combining 2 kg of NaCl obtained from the local grocery store, in the form of table salt, with 5 L of distilled water. When adding a solution to the field collection cups, approximately 2.5 cm layer of salt was first placed in the cup. This additional salt was placed in the cup to account for potential environmental water getting into the traps and diluting the salt solution from the initial concentration. The placement and collection of these traps followed the Canadian Food Inspection Agency's (CFIA) Survey Protocol: Invasive Alien Species Forestry Trapping (2017). Traps were placed at six sites for each location, left in the field for 4 weeks and collected during a single collecting event in August of 2017. Upon collection, specimens were removed from the collection cups, and the salt solution was poured directly into a new whirl pack, labeled, frozen at −20°C, and shipped to the laboratory packed in ice where they were then stored at −20°C until filtration.

**TABLE 1:** Lindgren funnel trap saturated salt solutions collected August 16, 2017, from six Wheatley and six Windsor Ontario locations

Volume (ml)	Site identifier
350	Wheatley—W1
300	Wheatley—W2
350	Wheatley—W3
300	Wheatley—W4
250	Wheatley—W5
250	Wheatley—W6
250	Windsor—B1
200	Windsor—B2
300	Windsor—B3
200	Windsor—B4
120	Windsor—B5
120	Windsor—B6

Note

Trap volume indicates the volume of solution in the traps at the time of collection. This volume varies due to environmental factors such as rain events and evaporation.

### 2.2 Filtration

All equipment necessary for trap fluid filtration were sterilized with 50% bleach or ELIMINase (Decon Labs) prior to use. Trap saturated salt solution was filtered through Nitrocellulose Mixed Ester membrane filters (pore size 1 µm, diameter 47 mm; Sterlitech). The filtration apparatus included a three‐port manifold connected to an EZ‐Stream vacuum pump (EMD Millipore) with magnetic filtration cups (Pall). Upon filtration, the membranes were stored at −80°C until use in eDNA extraction.

### 2.3 eDNA extraction

**FIGURE 1:**
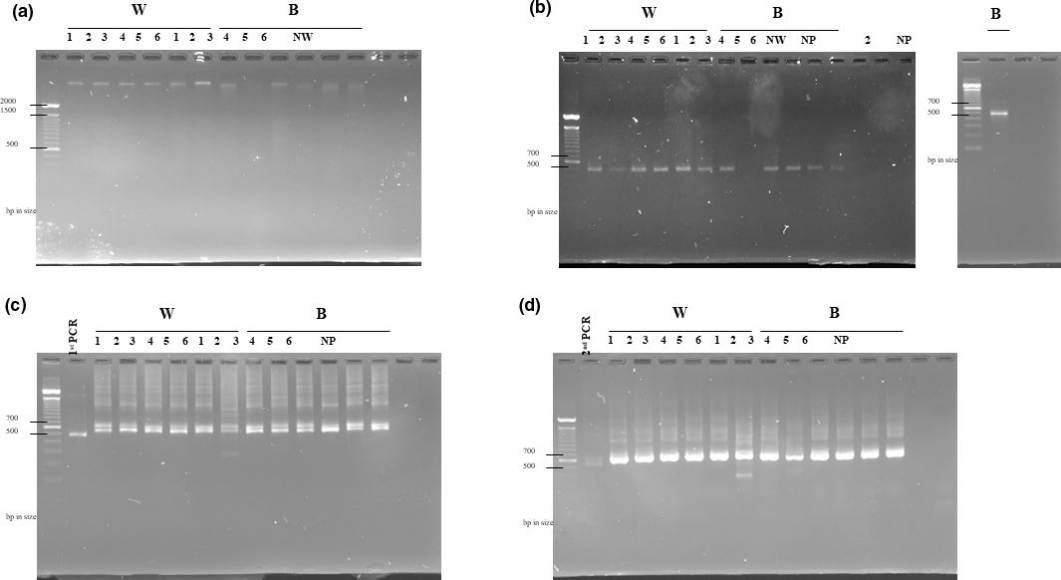
DNA agarose gel electrophoresis. (a) eDNA extracted from saturated salt solution in Lindgren funnel traps were separated on 1% agarose gel. (b) The first PCR to amplify the target COI gene region using each of extracted eDNA as a template and primers, mLepF1/RonMWASPdeg and C_LepFolR. Two amplification attempts were conducted using the same protocols for the sample in lane B2. The attempt on the far right of section B yielded positive results and was the thermocycling product used for the remainder of this study. (c) The second PCR and (d) the third PCR to attach Illumina adapter overhang nucleotide sequences to each of the first PCR amplicons and Illumina index primer sets to each of the second PCR amplicons, respectively. NW is a ddH_2_O negative control. NP is the PCR reaction without the template as a negative control for PCR contamination. W: Wheatley Ontario regional collection identifiers, B: Windsor Ontario regional collection identifiers. All DNAs in the gels were visualized by SYBR Safe DNA gel stain (Invitrogen)

eDNA was extracted using a CTAB method adapted from Dempster et al. (1999). Each membrane filter was cut into quarters using sterile razor blades and placed into individual 1.5 ml microcentrifuge tubes containing ~100 μl of glass beads (0.75–1 mm diameter). A volume of 500 μl of CTAB buffer (2% [w/v] polyvinylpyrrolidone, 1.4 M NaCl, 100 mM Tris‐HCL pH 8.0, 20 mM EDTA) prewarmed to 65°C was added to each tube followed by homogenization by Mini BeadBeater for 1 min (BioSpec Products). The tubes were then incubated at 65°C for 1 hr in a heat block with occasional gentle mixing by inversion. After incubation, 500 μl of 24:1 (v/v) chloroform: isoamyl alcohol was added to each tube and gently mixed by inverting for 1 min. The tubes were centrifuged at 13,000 *g* for 15 min, and the supernatants were transferred into new 1.5 ml microcentrifuge tubes. An equal volume of isopropyl alcohol and a half volume of 5 M NaCl were added to each tube, which were then mixed, placed at −20°C overnight, and centrifuged at 13,000 *g* for 15 min. The supernatants were discarded, and the pellets were washed in 500 μl of 70% (v/v) ethanol. The pellet was resuspended in 200 μl of ddH_2_O. After eDNA concentrations were determined by a NanoDrop spectrophotometer (Thermo Fisher Scientific), samples were separated on a 1% agarose gel electrophoresis (Figure 1a) to verify the length fragments of the DNA alongside a ddH_2_O negative control (NW). All eDNA extracts were stored at 4°C for immediate use or −20°C for short‐term storage before use in polymerase chain reaction (PCR).

### 2.4 Library preparation

**FIGURE 2:**
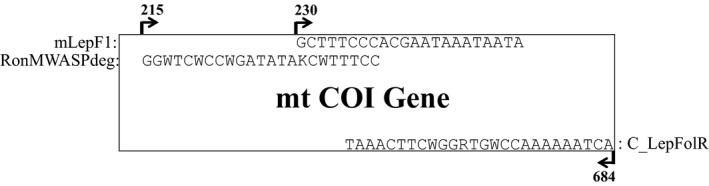
Positions of primers used in this study in relation to the Folmer COI‐5P animal barcode region

To amplify a 407‐nucleotide region of the animal DNA barcode region (COI‐5P), PCR was performed following the standard protocol developed by Ivanova et al. (2006). PCR reactions were set up in a total volume of 50 μl comprising 50–100 ng of each eDNA extract, 1× PCR Buffer, 1.5 mM MgCl_2_, 0.2 mM dNTP each, 0.2 μM primer each (mLepF1/RonMWASPdeg forward and C_LepFolR reverse primer; Figure 2), and 2 U Platinum Taq DNA Polymerase (Invitrogen). Primers were chosen to amplify a diverse set of taxa expected to be potentially present in the Lindgren funnel traps. The mLepF1 primer is a forward primer which has been shown to amplify a variety of insect taxa (Hajibabaei et al., 2006) and the RonMWASPdeg primer was used to amplify additional flighted insect species due to its degenerate nature (Smith et al., 2012). The reverse primers were a cocktail of two primers that resulted in an approximate amplified fragment of 407 nucleotides in length which were of suitable length for the Illumina MiSeq System (Hernández‐Triana et al., 2014). To facilitate sequencing library preparation, M13 (5′‐tgtaaaacgacggccagt‐3′) forward and M13 (5′‐caggaaacagctatgac‐3′) reverse nucleotide sequences were attached to the mLepF1 and RonMWASPdeg forward and C_LepFolR reverse primers, respectively. A thermocycling profile with an initial denaturation at 94°C for 2 min, 5 cycles of 94°C for 3 s, 45°C for 40 s, and 72°C for 30 s, followed by 35 cycles of 94°C for 30 s, 51°C for 40 s, and 72°C for 30 s, and a final extension at 72°C for 1 min. After the sizes of the PCR products were verified by agarose gel electrophoresis (Figure 1b) alongside a ddH_2_O negative control (NW) and a no template control (NP) to assess the potential contamination in reaction reagents, the amplicons were purified with AMPure XP magnetic beads (Beckman Coulter), following manufacturer's protocol, and quantified using a NanoDrop spectrophotometer (Thermo Fisher Scientific).

Sequencing libraries were completed by two PCRs to incorporate adaptors for downstream sequencing. Initially, to add Illumina adaptor sequence sets, PCR was conducted in 25 μl reaction volumes containing 15 ng of each purified COI‐5P amplicon, 0.2 μM each primer (Forward‐M13F: 5′‐TCGTCGGCAGCGTCAGATGTGTATAAGAGACAGTGTAAAACGACGGCCAGT‐3′ and Reverse‐M13R: 5′‐GTCTCGTGGGCTCGGAGATGTGTATAAGAGACAGCAGGA AACAGCTATGAC‐3′), and 12.5 μl of 2× KAPA HiFi HotStart Ready Mix (Roache Diagnostics). The cycling parameters consist of an initial denaturation at 95°C for 3 min, 25 cycles of 95°C for 30 s, 46°C for 40 s, and 72°C for 40 s, and a final extension at 72°C for 3 min. PCR products were verified on a 1% agarose gel alongside a no template control (NP) to assess the potential contamination in reaction reagents and purified using AMPure XP magnetic beads (Beckman Coulter) as per the manufacturer's protocol (Figure 1c). The amplicons were quantified using a NanoDrop spectrophotometer.

Next, a Nextera XT Index Kit (Illumina) was used to complete the first library preparation from the PCR products. A 50 μl reaction comprising 50 ng of purified COI‐5P/Illumina adaptor sequence amplicons, 5 μl each set of 10 μM concentration index primers, and 25 μl of 2× KAPA HiFi HotStart Ready Mix. A thermocycling reaction of 95°C for 3 min, 10 cycles of 95°C for 30 s, 55°C for 40 s, and 72°C for 40 s, and a final extension at 72°C for 3 min. The amplicons were verified on an agarose gel alongside a no template control (NP) to assess the potential contamination in reaction reagents and cleaned using AMPure XP magnetic beads (Beckman Coulter) using the manufacturer's protocol (Figure 1d).

### 2.5 High‐throughput sequencing

Sequencing was performed at the Genomics Facility, Advanced Analysis Centre, University of Guelph. Sequencing libraries were quantified with the Qubit dsDNA HS assay kit (Thermo Fisher Scientific), assessed for fragment size in a Bioanalyzer HS DNA Chip (Agilent), normalized using SequalPrep Normalization Kit (Thermo Fisher Scientific), and sequenced on an Illumina MiSeq System using a MiSeq reagent kit v3 (600 cycles). Each trap was processed on approximately 1% of the total capacity of the MiSeq run. Sequencing reads were demultiplexed and adapter trimmed with the MiSeq Reporter software, generating two paired‐end raw FASTQ files.

### 2.6 Sequence analyses

**FIGURE 3:**
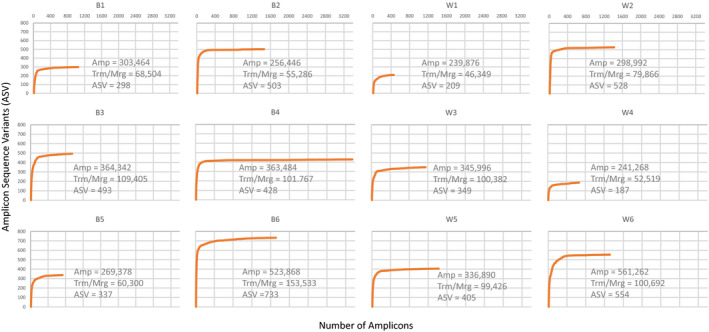
Log transformed rarefaction plots for each of the Lindgren funnel trap saturated salt solution samples. Amplicons with more than 10 reads were included. Numbers in the figure represent the total number of amplicons (Amp), the number of sequences after end trimming and paired‐end read merging (Trm/Mrg), and the final number of amplicon sequence variants (ASV)

The Geneious Prime (ver 2020.1.1; Biomatters, Ltd.) analytical software was used to obtain final merged paired‐end reads to assess the total number of comparable amplicons (Amp), merged sequences, and unique sequences or amplicon sequence variants (ASV). Analytical steps included quality trimming (>20 Phred) and amplicons with greater than 250 nucleotides were retained and merged using Geneious Prime. The resulting amplicons that were able to be paired were used for sequencing depth analysis to obtain the number of unique sequences or amplicon sequence variants (ASV; Figure 3). The number of ASVs was assessed using R (R Core Team, 2019—v. 3.6.1) and a custom R script (Appendix S1). ASVs with greater than 10 reads per unique sequence were used.

A parallel analysis was conducted using the MiSeq data and the analytical platform Multiplex Barcode Research And Visualization Environment (mBRAVE; http://mbrave.net/). Settings and libraries used to analyze the results from the high‐throughput sequencing can be found in Appendix S2. Results obtained from the mBRAVE platform were in the form of matches to existing data on the Barcode of Life Data System (Ratnasingham & Hebert, 2007), in the form of sequences and existing Barcode Index Numbers (Ratnasingham & Hebert, 2013). Where sequences did not match to BINs, a clustering analysis was conducted, and sequences were grouped into molecular operational taxonomic units (OTU) using a 2% sequence divergence threshold (Blaxter, 2004). All results from the mBRAVE analysis with more than 10 reads were used for discussions.

## 3 RESULTS

Final eDNA extracted from the 12 salt trap collections were successfully amplified. Amplicons with greater than 10 reads were used to create the ASV accumulation curves. These results showed that for all traps, a consistently high amplicon yield of between 239,876 and 561,262 sequences, but that the number of ASV was not proportionally consistent across the sampled sites (Figure 3).

The mBRAVE analyses provided matches to barcode records for metazoans with barcode sequence representation in the Barcode of Life Data (BOLD) system through BINs (Ratnasingham & Hebert, 2013). Sequences that did not match to a BIN were clustered using a 2% nucleotide divergence threshold and then matched to the closest sequence in BOLD (Table 2). Although the mBRAVE analyses did identify fungal taxa, these results were not evaluated further as they were not target taxa for this study and only represented less than 0.1% of amplicons for all collected biological samples. Additionally, there were only 16 fungal taxa identified with greater than 10 reads and only two of these (*Penicillium bialowiezense*, *Penicillium olsonii*) were identified to a species level, both of which are common taxa in the collection region. While some amplicons were matched to bacterial records (approximately 2% of all obtained amplicons), these represented common and nontarget taxa for this study and so these results were not evaluated further.

**TABLE 2:** mBRAVE analyses of the next‐generation sequencing MiSeq results

Run name	Number of reads	Number of reads Postfilter	BINs	OTUs
B1	112,389	98,512	181	71
B2	95,737	66,235	60	88
B3	148,417	136,932	104	98
B4	142,832	133,487	204	80
B5	98,912	87,579	253	68
B6	212,101	196,786	257	93
W1	85,673	69,995	63	115
W2	120,024	106,313	102	136
W3	140,695	128,002	133	129
W4	87,561	78,191	101	81
W5	138,630	126,103	152	95
W6	224,201	116,379	139	96

Note

The run name is the list of site identifiers for the collected traps. The number of reads is the total number of raw amplicons submitted to mBRAVE. The number of reads postfilter is the remaining reads after end trimming and quality filtering. BINs are the number of existing Barcode Index Numbers (BIN) in the BOLD System to which paired‐end quality trimmed sequences matched. Finally, OTUs are the number of molecular groupings based on a 2% clustering that was matched by similarity to the closest sequence in BOLD for reads that did not match to an existing BIN.

The remaining fungal and bacterial free dataset across all collections resulted in 1,656 BINs and 1,150 OTUs using a 2% clustering threshold. The OTUs were generated using amplicons not matched to BINs and represented approximately 12.6% of the total amplicons for all collections. Of these 1,150 OTU's there were 643 with greater than 10 reads, of which 400 did not possess any taxonomic information. The remaining 243 OTUs had varying levels of taxonomic identifications, with 75 identified to genus and 86 to species and only 118 OTUs of the target taxonomic group Arthropoda. In total, for the OTUs generated, there were 46 unique species and 69 unique genera with matches having a percent similarity ranging from 90% to 97%. There were no taxa identified to genera or species of concern from a Canadian regulatory perspective (List of pests regulated by Canada, 2020). As reliable identifications necessary for regulatory decision making were not obtained for OTU data, further analyses of these records were not conducted.

**FIGURE 4:**
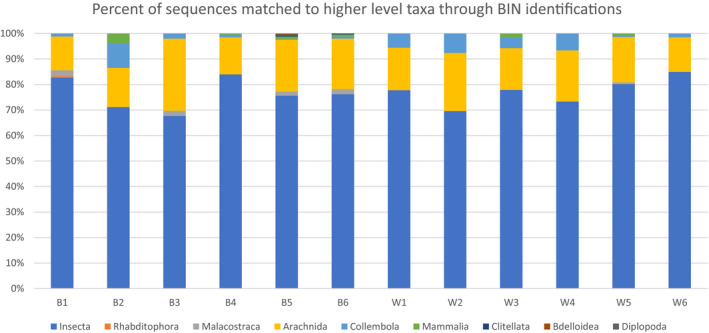
Higher‐level taxonomic identification of sequences using mBRAVE for all collected traps, six sites in Windsor Ontario (B1–B6) and six sites in Wheatley Ontario (W1–W6). Bar chart segments represent the percentage of unique sequences with greater than 10 reads identified to phyla (excluding fungal and bacterial sequences)

Molecular identifications through BINs for unique sequences, generated per collection site of greater than 109 reads, provided varying taxonomic levels. The main groups represented in the BIN analyses included chordates, noninsect arthropods, nonarthropod invertebrates, and insects. Insects accounted for 67%–86% of identifications across collected samples (Figure 4). This higher proportion of insects is expected as the biological collection traps were targeting insect taxa.

In total, 416,979 unique sequences with greater than 10 reads that were placed into BINs that represented 180 different binomial names (full list included in supplemental files). There were also 239,306 sequences that were matched to 161 unidentified BINs in 111 named genera. The sequences which had associated species names were representative of 19 different orders, 12 of which were insects (Blattodea, Coleoptera, Diptera, Ephemeroptera, Hemiptera, Hymenoptera, Lepidoptera, Neuroptera, Orthoptera, Psocodea, Thysanoptera, Trichoptera), with the target coleopteran taxa making up 38% (69/180) of identified species. There were 19 species identifications of note from our results, where all remaining species identifications obtained from our analyses were native in the collected region (Table 3).

**TABLE 3:** Identified taxa of interest from the quality filtered and trimmed paired‐end sequence data across all collections

Order	Family	Species	B1	B2	B3	B4	B5	B6	W1	W2	W3	W4	W5	W6	Note	Reference
Introduced species																
Coleoptera	Curculionidae	*Mecinus pyraster*	11												Introduced European weevil to combat spread of introduced plants (Plantago and toadflax)	Anderson et al. (2018)
Invasive and non‐native species																
Coleoptera	Curculionidae	*Xylosandrus germanus*	11								3,060	42	89	92	Invasive pest species	Ranger et al. (2016)
Coleoptera	Scarabaeidae	*Popillia japonica*												88	Invasive target wood‐boring Japanese beetle	Bragard et al. (2018)
Coleoptera	Curculionidae	*Anisandrus maiche*	1,340	411	552	955	443	464	1,924	1,152	2,352	978	4,182	2,845	Non‐native ambrosia beetle	Rabaglia et al. (2009)
North American pest species																
Diptera	Cecidomyiidae	*Vitisiella brevicauda*											233		North American grape pest	Gagné (2009)
Coleoptera	Curculionidae	*Sciaphilus asperatus*	15,027		233	20				472	982			2,171	North America raspberry pest	Levesque and Levesque (2017)
Hemiptera	Cicadellidae	*Empoasca fabae*				20									North American field agricultural pest	Maredia et al. (1998)
Hemiptera	Phylloxeridae	*Daktulosphaira vitifoliae*	294		4,064	957	11,935	5,038					126	238	North American viticulture pest	Granett et al. (2001)
Psocodea	Liposcelididae	*Liposcelis decolor*		15											North American stored product pest	Nayak et al. (2014)
European species established in North America																
Coleoptera	Staphylinidae	*Sepedophilus testaceus*												78	European species established in North America	Klimaszewski and Brunke (2018)
Hemiptera	Aphididae	*Therioaphis trifolii*											41	34	European species established in North America	Sunnucks et al. (1997)
Hymenoptera	Formicidae	*Lasius alienus*				10	34	37							European species established in North America	Maerz et al. (2005)
Isopoda	Trachelipodidae	*Trachelipus rathkii*						18							European species established in North America	Hornung et al. (2015)
Species not expected due to sampling methodology																
Carnivora	Procyonidae	*Procyon lotor*		30							13		79		Common raccoon	Rosatte (2000)
Rodentia	Muridae	*Mus musculus*		23										41	Common house mouse	https://www.gbif.org/species/7429082
North American species with multiple BINs																
Coleoptera	Coccinellidae	*Anatis labiculata*											626		BOLD:ACA6082, BOLD:AAF3857	Watson (1976)
Coleoptera	Mordellidae	*Mordellaria serval*	927		242	155	1,231	8,801						330	BOLD:ABX8545, BOLD:ABX3317, BOLD:AAU7283	https://www.gbif.org/species/133529744/verbatim
Isopoda	Philosciidae	*Philoscia muscorum*	2,608		1,785										BOLD:AAH4103, BOLD:AAH4104	https://www.gbif.org/species/2209507
Native biological control species																
Hemiptera	Anthocoridae	*Orius insidiosus*									32				Predator of pest arthropod species (Frankliniella occidentalis)	Funderburk et al. (2000)

Note

The numbers indicate the total number of sequences for each collection (B1–B6, W1–W6). The note and references contain justification for these species being of interest in biological surveys.

There were two species (*Popillia japonica* and *Daktulosphaira vitifoliae*) and one genus (*Lymantria*) listed as pests by the Canadian government (List of pests regulated by Canada, 2020). There were also two other notable invasive taxa not on the Canadian regulatory list, *Anisandrus maiche* and *Xylosandrus germanus* (Rabaglia et al., 2009; Ranger et al., 2016; Terekhova & Skrylnik, 2012; Table 3). In addition, there were four agricultural pests identified in our survey *Vitisiella brevicauda*, *Sciaphilus asperatus*, *Empoasca fabae*, and *Liposcelis decolor*. There were also four European species identified which have become established in North America (*Sepedophilus testaceus*, *Therioaphis trifolii*, *Lasius alienus*, and *Trachelipus rathkii*). There were two species, one native and another introduced, used in biological control management practices (introduced *Mecinus pyraster* and native *Orius insidiosus*).

Sequences present in the dataset also resulted in three matches to BINs with species names that have more than one associated BIN (*Anatis labiculata*, *Mordellaria serval*, *Philoscia muscorum*). There were also two species identified in our samples that were not expected to be present given the trapping methodology. In three traps (B2, W3, W5), there was sequence that matched to Raccoon (*Procyon lotor*), and in two traps (B2, W6) the common house mouse (*Mus musculus*) was identified.

## 4 DISCUSSION

Biosurveillance protocols have been a persistent research and monitoring tool to better understand the presence and distribution of species. Historically, these surveillance efforts used morphological identifications and were generally divided into two groups, targeted surveillance, and biodiversity assessments. With the advancement of molecular identification tools and DNA metabarcoding methodologies, the possibility of establishing biosurveillance protocols to simultaneously address both targeted and biodiversity surveys is now available.

Our resulting high numbers of unique high‐quality (Figure 1, Table 2) paired‐end reads for each of our traps provides evidence for the ability of the salt solution to maintain relatively high molecular weight DNA suitable for making species‐level identification where high‐quality reference sequence libraries exist. The high number of reads obtained from our mBRAVE analyses (Table 2) and the variation of matched results to sequences, gives us an indication that the preservation of the eDNA in the salt solution was not limited to one or few large specimens, but representative of many different taxa present in the solution. The large variety of taxa matched to reference sequences in the BOLD database include 341 taxonomic units as represented by species names and BINs in 252 genera, of which 26.9% were within the target group of Coleopterans. This further supports the efficacy of this protocol as an effective biosurveillance tool for Coleopteran species of regulatory concern.

Among the taxa identified using molecular evidence, there were six taxa categorized as either introduced, non‐native, invasive, or pest, within the survey region (Table 3). Four of these taxa were in the target group Coleoptera: *M. pyraster*, *X. germanus*, *A. maiche*, and *P. japonica*. *P. japonica* is on the Canadian government plant health list of species of regulatory concern and has been a known pest to native flora and agriculture for over 25 years (Allsopp, 1996). Although the recorded presence of this species in a known invaded range is not unexpected, the effective identification of *P. japonica* using this trapping and molecular protocol is useful in that the application of this biosurveillance protocol could aid in future species tracking and biocontrol review and management decisions.

With the successful amplification of both fungal and bacterial DNA in the protocols presented here, there is an opportunity to include different higher‐level taxa as targets using the salt trap medium and could be optimized with more taxon‐specific primers (Y. Milián‐García, R. G. Young, M. Madden, E. Bullas‐Appleton, & R. H. Hanner, 2020, in preparation). For instance, in field applications of biological control protocols, traps placed in the control zone followed by a metabarcoding methodology of samples, can provide survey data for large scale applications which can be used to assess the impact of the biological control measures (Milián‐García, Young, Madden, Bullas‐Appleton, & Hanner, 2020, in preparation). Our results contained amplicons identified within the same orders used by the biocontrol example studies referenced above, Rhabditida nematodes for Marianelli et al. (2018) and Hypocreales fungus for Benvenuti et al. (2019). This further provides support that the salt trapping followed by metabarcoding analyses outlined here could be effectively applied to survey both target pest specimen as well as introduced biocontrol agents.

A strength of the approach taken with this metabarcoding protocol using a salt trap solution is in the resulting molecular extract which can be used not only in the target study but the unused portion can also be archived in a cryopreserve for use in future studies. For instance, if the original study was not targeting a more recently identified pest taxa, there is opportunity to access cryopreserved extracts for use in these potential future studies and could benefit from a dedicated repository for extracts. These additional DNA extracts can be used for a variety of purposes, including additional high‐throughput sequencing and metabarcoding analyses using differing methods or targeted detection using a quantitative PCR protocol. This could be especially important in helping to identify native predators or pathogens for use in biocontrol of invasives like *P. japonica* and the protocols established by Marianelli et al. (2018) using Italian native biocontrol species.

Two other taxa on the CFIA list of regulated pests were also identified. *D. vitifoliae* is an introduced pest to Canada with severe impacts on grapevines and commercial grape operations. Management options for this pest have centered around the production of resistant grapevine strains, and the monitoring of *D. vitifoliae* distributions can assist in the effective planting strategies for the grape industry (Granett et al., 2001). In addition, the sequences obtained using a trapping and metabarcoding protocol could provide insight into the population structure of *D. vitifoliae* and contribute to the elucidation of the distribution patterns of this pest. The final taxon of regulatory concern identified in our work was the genus *Lymantria*. There are numerous species of concern among *Lymantria*, including the invasive *L. dispar*. Although specific efforts to complete a DNA barcode library for all species in this genus have been underway, the lack of resolution in matching the amplicons for this study to a specific species indicates that further efforts are needed to populate this library and make it publicly available (deWaard et al., 2010).

There were also several species identified which are of interest due to potential impacts on native and agricultural flora and fauna but are not on the Canadian plant health list of regulated species. The detection of one such species, *A. maiche* is notable for two reasons. Firstly, it was the only taxa identified across all collections. Secondly, it is a recently introduced species to North America (Rabaglia et al., 2009) but has yet to be reported as present in Canada in the peer‐reviewed literature, and is not currently on the CFIA plant pest species list. *A. maiche* was first identified in North America in Pennsylvania in 2005 (Rabaglia et al., 2009). *A. maiche* is an Asian species of wood‐boring bark beetle which predominantly infests damaged or weakened trees (Ranger et al., 2016; Terekhova & Skrylnik, 2012). It is destructive to a variety of trees and cultured ornamentals and once introduced into suitable habitat it quickly establishes and becomes abundant (Rabaglia et al., 2009; Ranger et al., 2016). The prevalence of *A. maiche* in our samples is consistent with the notion that this species is quick to establish and become abundant in the invaded range.

With the successful application of a salt suspension solution outlined here, it seems prudent to replace current protocols that utilize insect trap solutions that are expensive, hazardous to human and animal health, and are difficult to work with. For example, while the use of ethanol is effective in its ability to preserve specimens for downstream molecular and morphological analyses, it does present several challenges (deWaard et al., 2019). One of these challenges is the high evaporation rate, and this is especially true in warm weather climates where frequent monitoring and top‐ups of suspension fluid are required. Although in warm climates the salt solution will still be subject to evaporation, it will be far less of a problem and require less regular maintenance of traps. When evaporation occurs, the salt will remain in the collection vessel, and the simple addition of distilled water is all that would be necessary to maintain the traps until collection. Further, when trap solutions are needing to be exchanged, the supplies, distilled water, and table salt, to accomplish this are easily accessible and inexpensive and can often be obtained through local vendors, whereas this is not usually the case for ethanol. This is especially true in remote locations where local regulations and laws limit the purchase and use of alcohol‐based substances (Gilchrist, 2004). Finally, ethanol is a flammable substance and, as such, is expensive to ship as a dangerous good. Our results indicate that the use of a saturated salt solution has effectively addressed each of these stated concerns.

## 5 CONCLUSION

The methods outlined here constitute a highly effective protocol for the molecular biosurveillance of wood‐boring beetles. We have established that the use of a saturated salt solution as an effective alternative to other commonly used insect trap suspension solutions such as ethanol and propylene glycol. Our results were able to detect a wide range of taxa in the sampled region, including several introduced and pest taxa. The adoption of this highly effective protocol is recommended as a supplement for any small terrestrial arthropod trapping collections.

## CONFLICT OF INTEREST

None declared.

## AUTHOR CONTRIBUTIONS

**Robert G. Young:** Conceptualization (equal); Data curation (equal); Formal analysis (equal); Methodology (equal); Validation (equal); Visualization (equal); Writing‐original draft (equal); Writing‐review & editing (equal). **Yoamel Milián‐García:** Data curation (equal); Methodology (equal); Writing‐review & editing (equal). **Jaeju Yu:** Data curation (equal); Formal analysis (equal); Investigation (equal); Writing‐review & editing (equal). **Erin Bullas‐Appleton:** Conceptualization (equal); Methodology (equal); Project administration (equal); Writing‐review & editing (equal). **Robert Hanner:** Conceptualization (equal); Funding acquisition (equal); Investigation (equal); Methodology (equal); Project administration (equal); Writing‐review & editing (equal).

## Supporting information

Appendix S1‐S2

## Data Availability

Original fastq datasets and intermediate datasets, and final results are archived and freely accessible at the University of Guelph Agri‐environmental research data repository Dataverse (https://dataverse.scholarsportal.info/dataverse/ugardr). Files include: Compressed file with all raw MiSeq sequencing results; Compressed file with Geneious merging results; Full ASV results from Geneious data and R script; CSV file with the accumulated results from the mBRAVE system.
