# Supplementary material for: Biosurveillance for invasive insect pest species using an environmental DNA metabarcoding approach and a high salt trap collection fluid
Source: Ecol Evol. 2021 Jan 28;11(4):1558–69. doi: 10.1002/ece3.7113 (PMC7882945; doi:10.1002/ece3.7113)
Supplement: Supplementary file 1 — Appendix S1‐S2 [file ECE3-11-1558-s001.docx]

**Appendix 1**

# Written by Rob Young at the University of Guelph in Ontario Canada, February 2020

#****************Main program section************************************

# prompting to choose the folder location of the working directory with the input file to run the program

print("Choose the folder location where your input files are located")

Work_loc<-choose.dir()

#set the format for the date for all operating systems

Sys.setlocale("LC_TIME", "C")

# Current Date - for file naming use

date <- sub("-", "", sub("-", "", Sys.Date()))

start_time=Sys.time()

print(paste("Start time... ",start_time))

#Get all files located in this folder.

#Initiating the list of files in the folders

path_list <- list.files(path=Work_loc, pattern = "*[.][Ff][Aa][Ss]$", full.names = TRUE)

file_list <- list.files(path=Work_loc, pattern = "*[.][Ff][Aa][Ss]$")

file_list_old <- file_list

file_name <- sub("\\..*","",as.vector(file_list))

for(h in 1:length(file_name)){

# load in the data file in

Seq_file<-data.frame(read.table(path_list[h], header=F,sep="\t",dec=".", quote="",comment.char = "", fill =TRUE))

#*****************************************************************************

#This section is taking the fasta file and formatting it in to columns to create the input file for the rates iteration program

#taking the read in file and changing from Fasta to tab delimited

Header0 <- Seq_file[seq(from = 1, to = nrow(Seq_file), by = 2), 1]

Sequence <- Seq_file[seq(from = 2, to = nrow(Seq_file), by = 2), 1]

#*****************************************************************************

list_of_num_of_unique_reads<-as.character(Sequence)

list_of_num_of_unique_reads<-table(list_of_num_of_unique_reads)

list_of_num_of_unique_reads<-sort(list_of_num_of_unique_reads)

rownames(list_of_num_of_unique_reads)<-NULL

list_of_num_of_unique_reads <- list_of_num_of_unique_reads[list_of_num_of_unique_reads>10]

list_of_num_of_unique_reads<-t(as.data.frame(c(file_name[h],list_of_num_of_unique_reads)))

#Writing the Seq_tabletwo column matrix to file using the new line character as a separater which will then put each entry on a new line and make it fasta format

write.table(list_of_num_of_unique_reads,file=paste0(Work_loc,"/Output.txt"), na="", row.names=FALSE, col.names=FALSE, quote = FALSE,sep="\t", append=TRUE)

print(paste0("In the loop number ", h, " at the end of ", file_name[h]))

}

# load in the data file in

final_OTU_list<-data.frame(read.table(paste0(Work_loc,"/Output.txt"), header=F,sep="\t",dec=".", quote="",comment.char = "", fill =TRUE))

final_OTU_list<-t(final_OTU_list)

#Writing the Seq_tabletwo column matrix to file using the new line character as a separater which will then put each entry on a new line and make it fasta format

write.table(final_OTU_list,file=paste0(Work_loc,"/Output.txt"), na="", row.names=FALSE, col.names=FALSE, quote = FALSE,sep="\t", append=FALSE)

**Appendix 2**

Settings and libraries used for the Multiplex Barcode Research And Visualization Environment (mBRAVE; <http://mbrave.net/>) when analyzing the MiSeq data collected from Lindgren funnel traps in Southern Ontario.

**Settings**

Trimming

Trim Front – 40bp

Trim end – 40bp

Trim length – 450bp

Filtering

Min QV – 20

Min length – 350bp

Max bases with low QV – 4%

Max bases with ultra low QV – 1%

No pre-clustering

ID distance threshold – 2%

Exclude from OTU threshold – 3%

Minimum OTU size – 5

OTU threshold – 2%

Assembler min overlap – 20bp

Assembler max substitutions – 5bp

**System Libraries** (All libraries used were last updated April 12, 2020)

| **Code** | **Name** | **Sequences** | **BINs** | **Species** |
| --- | --- | --- | --- | --- |
| SYS-MBRAVEC | System Reference Library - Standard Contaminants Based on Reagent Production | 2,225 | 75 | 85 |
| SYS-HUMC | System Reference Library - Human Contamination Check | 17 | 1 | 1 |
| SYS-CRLINSECTA | System Reference Library for mBRAVE ID Engine - Insecta | 695,377 | 509,004 | 207,439 |
| SYS-CRLNONINSECTARTH | System Reference Library for mBRAVE ID Engine - Non-Insect Arthropoda | 81,893 | 62,021 | 24,210 |
| SYS-CRLNONARTHINVERT | System Reference Library for mBRAVE ID Engine - Non-Arthropoda Invertebrates | 74,724 | 45,282 | 30,712 |
| SYS-CRLCHORDATA | System Reference Library for mBRAVE ID Engine - Chordata | 96,361 | 49,306 | 38,007 |
| SYS-CRLAVES | System Reference Library for mBRAVE ID Engine - Aves | 9,812 | 6,786 | 5,789 |
| SYS-CRLBACTERIA | System Reference Library for mBRAVE ID Engine - Bacteria COI | 9,747 | 2,113 | 2,066 |
| SYS-CRLFUNGI | System Reference Library for mBRAVE ID Engine - Fungi COI | 2,196 | 590 | 565 |
